# A possibilistic framework for constraint-based metabolic flux analysis

**DOI:** 10.1186/1752-0509-3-79

**Published:** 2009-07-31

**Authors:** Francisco Llaneras, Antonio Sala, Jesús Picó

**Affiliations:** 1 Instituto de Automática AI2, Universidad Politécnica de Valencia, Camino de Vera s/n, 46022, Spain

## Abstract

**Background:**

Constraint-based models allow the calculation of the metabolic flux states that can be exhibited by cells, standing out as a powerful analytical tool, but they do not determine which of these are likely to be existing under given circumstances. Typical methods to perform these predictions are (a) flux balance analysis, which is based on the assumption that cell behaviour is optimal, and (b) metabolic flux analysis, which combines the model with experimental measurements.

**Results:**

Herein we discuss a possibilistic framework to perform metabolic flux estimations using a constraint-based model and a set of measurements. The methodology is able to handle inconsistencies, by considering sensors errors and model imprecision, to provide rich and reliable flux estimations. The methodology can be cast as linear programming problems, able to handle thousands of variables with efficiency, so it is suitable to deal with large-scale networks. Moreover, the possibilistic estimation does not attempt necessarily to predict the actual fluxes with precision, but rather to exploit the available data – even if those are scarce – to distinguish possible from impossible flux states in a gradual way.

**Conclusion:**

We introduce a possibilistic framework for the estimation of metabolic fluxes, which is shown to be flexible, reliable, usable in scenarios lacking data and computationally efficient.

## Background

Systems biology states that, in order to quantitatively understand and predict the cell behaviour, its constitutive components and their interactions must be studied as a whole system [1,2]. Metabolic networks are a paradigmatic example of this aim because, even incomplete as they may be, they are the best characterized cellular networks [3]. In recent times, the information embedded in metabolic networks is being used to assemble constraint-based models under the pseudo steady-state assumption, thus not requiring the knowledge of kinetic parameters, which are still rarely known [3,4]. Constraint-based models allow the calculation of the possible metabolic states or "behaviours" that can be exhibited by the cell; however, they do not predict which of these are likely under given circumstances. One approach to perform these predictions is flux balance analysis (FBA), which is based on the assumption that cell behaviour has evolved to be optimal in a certain sense [5,6]. It has been shown that FBA is able to predict the actual fluxes [7-9], but this requires to identify which are the relevant objectives for different conditions [7,10]. As an alternative, one could perform a metabolic flux analysis (MFA) which, generally speaking, is the exercise of estimating the fluxes shown by cells by combination of a constraint-based model and the set of available experimental measurements.

In order to estimate the intracellular fluxes, traditional metabolic flux analysis (TMFA) employs only measurements of uptake and production rates (i.e. influxes into and outfluxes from cells) that are stoichiometrically balanced [11]. This purely stoichiometric approach has some limitations, but most of them can be overcome with simple extensions, as it will be shown below.

One typical difficulty to be tackled by MFA is that the available measurements may be insufficient to estimate the intracellular fluxes, particularly in large-scale networks, because there may be different flux distributions compatible with the available measurements. To face this situation, intracellular information obtained from stable isotope tracer experiments has been incorporated in many studies (13C-MFA) [12-14]. Yet, data from isotope tracer experiments will not be considered in this work. Instead, we follow a constraint-based modeling approach, in the sense that we do not attempt necessarily to predict the actual fluxes with precision, but rather to distinguish "most possible" from "impossible" flux states, based on a suitable definition of "possibility", a constraint-based model and the *available *measurements, which in most cases do not include isotopic data.

Another option to face a lack of measurements is the use of some rational hypotheses to chose one flux distribution among those that are compatible with the measurements. For instance, Nookaew et al. have proposed to estimate the intracellular fluxes based on the assumption that cells are likely to use as many pathways as possible to maintain robustness and redundancy [15]. Related hypotheses have been formulated using the concept of elementary modes [16,17]. The assumption of optimal cell behavior typically used in FBA could be also used (e.g. [7]). It will be shown that the methodology we propose is able to detect these flux distribution that are equally possible (or similarly possible), but for the sake of simple exposition we will not use any hypothesis herein. However, the possibilistic framework might be extended to incorporate hypotheses, as discussed in the conclusions section.

In this context, the paper discusses the use of a possibilistic framework for metabolic flux analysis.

Uncertainty, lack of measurements and model imprecision will be handled introducing the notion of "degree of possibility". Then, an efficient optimization-based approach will be employed to query the most possible fluxes and their possibility distributions. The methodology is based on a re-interpretation of the consistent causal reasoning paradigm [18] as an equivalent problem of feasibility subject to equality and inequality constraints; preferences under uncertain knowledge are incorporated by transforming the feasibility problem into a linear optimisation one, which may be interpreted in possibilistic terms. The optimisation approach to logic reasoning has been previously explored by the research group to which the authors belong in [19-21], and this paper applies it to MFA.

The main features of the possibilistic framework introduced in the paper are the following: (i) it is based on a constraint-based model and not only on stoichiometric balances, (ii) it considers measurements uncertainty in a flexible way (e.g. non-symmetric error or a band of uncertainty due to *systemic *error) and (iii) even model imprecision, (iv) it provides possibility distributions (and intervals) which are more informative than point-wise estimations when multiple flux values might be reasonably possible, (v) it is reliable even if only a few fluxes are measurable, (vi) it has the ability to detect, and handle, inconsistencies between measurements and model, and furthermore (vii) with high computational efficiency.

The structure of the paper is as follows: preliminaries on possibility, optimization and metabolic flux analysis are first addressed. Then, the basics of Possibilistic MFA and some refinements are discussed; the framework is illustrated with simple examples and a well-know model of *C. glutamicum*. The paper is closed with a summary and a discussion on future work.

### Preliminaries: possibility and optimization

In an abstract ideal situation, many estimation problems in science and engineering can be cast as estimating some decision variables *δ *given the known values of a set of other ones *m *(possibly, measurements) and a model expressed as a set of equality and inequality constraints (involving decision variables, measurements and some model parameters). Then, the valid estimations will be the feasible solutions of a constraint satisfaction problem [22,23].

However, in many practical cases, the measurements are imprecise and the model parameters and constraints are also not accurate, so real data violates them. This is the reason why most real-life models should include uncertainty. The most basic representation of uncertainty would be giving interval values to measurements and model parameters. Refinements of the uncertainty representation give rise to probabilistic [23-25] and possibilistic [26-28] frameworks.

Probabilistic frameworks have an underlying interpretation in terms of the frequency in which some flux conditions appear; on the other hand, possibilistic frameworks measure the degree of compliance (*consistency*) of some decision variables with some (soft) modeling constraints. In this sense, the basic assumptions of both paradigms of inference under uncertainty are different.

In the following subsections the possibilistic framework will be described. Afterwards, this section of preliminaries will be closed discussing the relationship between probability and possibility and justifying the use the possibilistic framework.

#### Soft constraint satisfaction problems: a possibilistic approach

As explained above, the possibilistic framework is the chosen representation for the problem under study, following the ideas in [29], where possibilistic constraint satisfaction problems (CSP) are presented. There, the authors introduce constraints which are satisfied to a degree, transforming the feasibility/infeasibility of a potential solution into a *gradual *notion: given a CSP with multiple solutions *δ *∈ Δ (where Δ denotes the search space over which feasible values for the decision variables will be searched), a function *π *: Δ → [0, 1] was suggested in order to represent preference or priority as a "consistency degree". The meaning of *π*(*δ*) = 1 would indicate that *δ *is in full agreement with the model and measurement constraints; the meaning of *π*(*δ*) = 0 indicates that *δ *is in "absolute, total contradiction" with the problem constraints, and never should be considered a feasible value. Intermediate values would denote values of decision variables which "somehow mildly" violate the problem constraints but could be considered "partially possible" from the "practical" knowledge of the "expert" modeller who defined *π*. The higher the value of *π*(*δ*), the higher the accordance with the problem constraints should be (subjectively interpreted as a higher "possibility" of the decision variable choice *δ*). Given the here outlined subjective meaning of *π*, it is denoted in literature as *possibility distribution*. The possibilistic calculus [27,29] refers then, to computations with possibility distributions from a series of axioms. Basic ideas on it will be outlined below in this section. A simple example now illustrates the basic idea.

##### Example

Consider a flux balance {*f*_1 _= *f*_2_}, stating equality between two flows, *f*_1 _and *f*_2_, supposedly measured in a biological or chemical reaction. The measurements *m*_*a *_= (5, 7) and *m*_*b *_= (5, 5.1) are infeasible, whereas *m*_*c *_= (5, 5) is feasible. However, it seems clear that the subjective "possibility" of *m*_*b *_is higher than that of *m*_*a*_; *m*_*b *_can be thought to be quite reasonable in practice due to measurement errors.

The idea can be easily formalised for further computations by defining a possibility distribution, for instance, . In this way, potential solutions can be ranked: *π*(*m*_*a*_) = 0.018, *π *(*m*_*b*_) = 0.99 and *π*(*m*_*c*_) = 1. The search space in which to define the possibility, Δ, could be defined as, say, Δ = {(*δ*_1_, *δ*_2_)|0 ≤ *δ*_*i *_≤ 10}.

Usually, the function *π*(*δ*) is built by "conjunction" of possibility functions of individual relations *π*_*i*_(*δ*_*i*_) (expressing user-defined preference or priority on each individual constraint, in many cases in a problem-dependent way). Such conjunction will be latter discussed in this section. The best CSP solutions are defined to be those which satisfy the global problem to the maximal degree.

In this way, once the user has defined such function expressing how a particular combination of system variables is "consistent" with its model, the basic idea on possibilistic calculus is, given a subset of the system variables (assumed as known or measured), estimate the "most possible" values of all the remaining variables via an optimization problem. The close relationship between possibilistic calculus and optimisation is discussed in the subsection below.

#### Possibility theory

The basic building block of possibility theory is a user-defined possibility distribution *π *: Δ → [0, 1]. This defines the possibility of each "point" *δ *in Δ. A consistent problem formulation is defined to be the one in which there exists at least one point with possibility equal to one.

The second building block are events, formally defined as subsets of Δ, in order to address problems such as, in the above example, determining the possibility of event *A *= {(*f*_1_, *f*_2_) ∈ Δ | 0 ≤ *f*_1 _≤ 3, 4 ≤ *f*_2 _≤ 10}.

##### Possibility calculus as optimization

By definition, the possibility of an event *A *(subset of Δ) is computed via:

(1)

and, obviously, given two events *A *and *B*, *A *⊂ *B *entails *π*(*A*) ≤ *π*(*B*).

Hence, possibility computations are optimisation problems (Cf. with probability computations, which are integration problems).

For a multidimensional Δ = Δ_1 _× Δ_2_, *δ *= (*δ*_1_, *δ*_2_) ∈ Δ, the *marginal *possibility distribution of *δ*_1 _is defined as:

(2)

i.e., the possibility of the event {*δ*_1 _= }.

##### Optimization as possibility calculus

Conversely, consider a cost function *J *: Δ → *R*^+ ^(*i.e*., verifying *J*(*δ*) ≤ 0 for all *δ *∈ Δ), so that there exists *δ*_0 _∈ Δ such that *J*(*δ*_0_) = 0. Then, a consistent possibility distribution may be defined on Δ via:

(3)

and the possibility of an event *A *is given by replacing the possibility definition (3) in (1), resulting in:

(4)

In the next sections, abusing the notation, an event *A *will be usually described by a set of constraints on the decision variables *δ*.

In this way, numeric constrained optimisation problems may be subjectively interpreted in possibilistic terms: the cost *J*(*δ*) will be interpreted as the log-possibility of *δ *and, by definition, unfeasible values of decision variables will be assigned zero possibility.

Let us now review some other relevant definitions and issues in possibilistic calculus.

#### Necessity

In order to assert that an event *A *is *necessarily *true (in our context, that all problem solutions belong to *A*), saying that *A *is "possible" may be not enough: it must also be true that the complementary event "not *A*" is not possible. This motivates the introduction of a necessity measure:

(5)

In a binary setting, all solutions belong to a subset *A *if and only if *π*(*A*) = *N*(*A*) = 1; there exist solutions in *A *(and solutions outside *A*) if *π*(*A*) = 1 but *N*(*A*) = 0, and there are no solutions in *A *if *π*(*A*) = 0.

Extending the measures *π*(*A*), *N*(*A*) to [0,1] provides a natural gradation of such concepts: *π*(*A*) = 0.95, *N*(*A*) = 0.1 would indicate that there are very possible solutions in *A*, but not all of them are in there (there are solutions with possibility 1-0.1 = 0.9 outside *A*).

#### Interactivity and possibilistic conjunction

The possibilistic analog to statistical independence is the non-interactivity.

If the joint possibility of two variables Δ = Δ_1 _× Δ_2_, *δ *= (*δ*_1_, *δ*_2_) ∈ *Δ *can be expressed as the product of two univariate ones:

(6)

then variables *δ*_1 _and *δ*_2 _are said to be non-interactive. In that way, given an event *A*_1 _⊂ Δ_1 _and an event *A*_2 _⊂ Δ_2_, it is straightforward to prove that:

(7)

which can be read as "the possibility of event *A*_1 _*and *event *A*_2 _is the product of the individual possibilities when the events relate non-interactive variables", interpreting, as usual in literature, set intersection as a linguistic conjunction.

Under non-interactivity assumption, if the possibility is defined as the logarithm of a cost index (3), the product (6) gets transformed into a sum:

(8)

On the following, given individual cost indices *J*_1_(*δ*_1_), *J*_2_(*δ*_2_), etc. relating to different constraints, the above expression (8) will be the one used in most cases to define a possibility distribution in the product space. In this way, we are interpreting the possibilistic conjunction operator in [29] as an algebraic product of possibilities, i.e., stating an underlying non-interactivity assumption between different constraints. Note, however, that the interactivity assumption is not always intuitively needed. In the other extreme (total interactivity: variables *δ*_1 _and *δ*_2 _fully "correlated", for instance equal), we would have: *π*(*A*_1 _∩ *A*_2_) ≤ max(*π*(*A*_1_), *π*(*A*_2_)), which would suggest the maximum possibility as the conjunction operator when two events affect exactly the same decision variables. In between those two extremes, other choices may be also possible (*T *-norm operators [30]).

#### Conditional possibility

The possibilistic analog to conditional probability is conditional possibility.

Consider an event *B *with nonzero possibility. A quotient definition for conditional possibility of an event *A *given event *B *will be used in this paper:

(9)

In this way, given a (multivariate) possibility distribution *π*(*δ*), the conditional possibility can be computed as:

(10)

so, if the possibility distribution is actually the exponential of a cost index, we get:

(11)

that is, computing the possibility by subtracting the cost associated to event *B *from the cost of any of its subsets.

In order to get a conditional possibility distribution of a variable *δ*, we assume event *A *being an individual point δ*, getting:

(12)

That is, the conditional distribution can be obtained by dividing the possibility distribution function for all points in a set by the maximum possibility of them, i.e., normalising the possibility distribution on a restricted conditioning domain *B *to a maximum equal to one.

The conditional definitions allow for an analogy to Bayesian inference: if we assume that *B *is actually certain (whatever the *a priori *possibility *π*(*B*) was), then conditional possibility may be understood as an *a posteriori *possibility.

#### Possibility versus probability

Both possibility theory and probability theory are frameworks for handling uncertainty in constraint satisfaction problems. Basically, a subjective interpretation would assign high possibility to events with high probability. Hence, in a first approximation, user-defined probabilities and possibilities should be related by an implicit monotonically increasing function. Possibility-necessity measures have also been linked to imprecise probabilities [31]. However, once aggregation takes place (via sums and integration in probability, via maximisations in possibility), although the subjective interpretation might be considered similar, there is no longer an implicit function relating probability and possibility. For further discussion on possibility, probability, and other uncertain reasoning frameworks, and their interrelations, the reader is referred to [31-33].

Ideally, probabilistic results would be preferable (to confidently assert that, e.g., 95% of cases a flux estimate will lie in a particular interval). However, there are some drawbacks: (i) exact probabilistic inference under equality and inequality modeling constraints is computationally hard (multivariate integration on irregular sets) (ii) some of the *a priori *Bayesian probabilities are in practice rough user-given estimates, (iii) some of the assumptions (linearity of transformation, Gaussian distributions) may not hold in practice, and (iv) there may be some uncertainty in the model parameters or in the model probabilities. Thus, as practical use of probability does not fully adhere to the theoretical assumptions, its results should be interpreted with some flexibility. As this work will discuss, the proposed possibilistic framework is much less demanding computationally (using optimization instead of integrals, so large-scale cases become tractable) and gives similar results to the probabilistic approach in realistic cases.

The objective of the next sections is to set up a possibilistic framework for efficient computations in metabolic flux analysis.

### Preliminaries for metabolic flux analysis

In biology, the metabolism of living cells is usually represented by a metabolic network encoding the elementary biochemical reactions taking place within the cell [3]. These metabolic networks can be translated to a matrix **N**, where rows are the *m *internal metabolites and columns the *n *reactions. Assuming that these metabolites are at steady state, mass balances can be formulated as follows [11]:

(13)

where **v **= (*v*_1_, *v*_2_,...,*v*_*n*_)^*T *^is the *n*-dimensional vector of metabolic fluxes.

Hence, a (steady-state) flux vector **v **represents the metabolic state of the cells at a given time, without any information on the kinetics of the reactions; it shows the contribution of each reaction to the overall metabolic processes of substrate utilization and product formation. Notice that as typically *n *is larger than *m*, the system (13) is underdetermined, i.e. there is a wide range of stoichiometrically-feasible flux vectors. Assuming now that some fluxes in **v **have been measured (denoted as *v*_*m*_), while the rest remain unknown (denoted as *v*_*u*_), equation (13) can be rearranged as follows:

(14)

As measurements are imprecise in practice, such measurement imprecision can be incorporated as constraints:

(15)

where **e**_**m **_represents measurements errors and  represents the actually measured flux value. In our approach, the measurement uncertainty is translated into an a-priori possibility distribution for **e**_**m **_from sensor characteristics. Other approaches consider different choices, as discussed below.

At this point, Traditional metabolic flux analysis (TMFA) can be defined as the estimation of the flux vector satisfying (14) and compatible with the measurements (15). In particular, TMFA can be formulated as a two step procedure [34,35]: (I) analyze measurements consistency (and detect gross errors) using chi-square tests, and (II) solve a least squares problem to estimate the actual flux vector **v**:

(16)

where it is assumed that **e**_**m **_are distributed normally with a mean value of zero and a variance-covariance matrix **F**.

Since all the constraints are linear *equalities*, the analytic solution of this minimization problem can be obtained, resulting in the expressions to estimate **v**_**u **_and **v**_**m **_that are typically seen in literature (e.g. [11,36]). However, with this formulation TMFA has some important limitations: (i) irreversibility constraints, or any other *inequality *constraints, cannot be considered, (ii) measurement errors are assumed to be normally distributed, (iii) it only provides unique-valued flux estimation, and (iv) it needs a high number of measurable fluxes to be of use – system (14) has to be determined and redundant [37].

Several alternatives have been suggested to face those limitations (Table 1). Quadratic programming solves the least squares problem (16) allowing to include irreversibility constraints (LS-MFA), but inherits the rest of drawbacks (and the chi-square tests may lose validity). The flux spectrum approach (FS-MFA) follows an interval approach to overcome the limitations mentioned before, but its estimations tend to be conservative because only lower and upper bounds are used to represent measurements uncertainty [38,39]. Monte Carlo has been also used in the context of 13C-MFA (e.g. [40-42]), but rarely in absence of isotopic data. Moreover, sometimes it has been used incorrectly: Monte Carlo cannot be performed just solving a quadratic programming problem for each simulated set of measurements, because this introduce a bias on the results. Anyway, the major drawback of Monte Carlo is its high computational cost, which restricts its use for large metabolic networks as an impractical number of samples is required to assess probabilities within a reasonable accuracy.

**Table 1:** Features of Possibilistic MFA and alternative approaches

Feature	TMFA	LS-MFA	FS-MFA	Monte Carlo	PMFA
Considers irreversible reaction		x	x	x	x
Usable in scenarios lacking measurements			x	o	x
Includes a check of consistency	x	-	-	o	x
Flexible description of measurements errors			-	x	x
Richer estimations (not only point-wise)			-	x	x
Computational efficiency	x	x	x		x

Possibilistic MFA (PMFA) is compared with four approaches for metabolic flux analysis, Traditional MFA (TMFA), MFA as a constraint least-squares problem (LS-MFA) and the flux spectrum approach (FS-MFA). Legend: (x) provided feature, (-) partially provided feature and (o) potentially provided feature.

In the following sections we introduce a possibilistic framework for MFA that brings several interesting features: (i) it overcomes all the mentioned limitations of TMFA, (ii) has the ability to detect, and handle, inconsistencies between measurements and model, and furthermore (iii) with high computational efficiency.

## Results and discussion

### Possibilistic MFA: problem statement

In this section the possibilistic framework for MFA flux estimations is discussed. First, we define a set of time-invariant constraints derived from the metabolism being modelled. Then we incorporate the constraints imposed by the measured fluxes, representing its uncertainty, by means of auxiliar slack decision variables and a cost index. In this way, the notion of "degree of possibility" is incorporated. Finally, it will be shown how (linear) optimisation problems will be able to settle queries about the most possible fluxes, the possibility distributions, etc.

#### Model-based constraints

First, let us define a set of invariant constraints that every steady-state flux vector must satisfy; they do not depend on environmental conditions, do not change through evolution, etc. [3]. In this work this *model-based constraints*, denoted as , will be the stoichiometric relationships (13) and irreversibility constraints, described by means of inequalities:

(17)

where **D **is a diagonal *nxn*-matrix with *D*_*i, i *_= 1 if the flux *i *is irreversible (otherwise 0).

Other model-based constraints can be defined in an analogous way. For instance, elementary balances or degree of reduction balances might be incorporated into (17) as additional constraints [11]. It may be also possible to add constraints based on standard Gibbs free energy changes [43,44] or extracellular metabolites concentrations [45].

#### Incorporating the measurements

Estimating the non-measured fluxes would amount for solving the above equations (17), where some of the elements in vector **v **are measured (**v**_**m**_). However, this simple approach will be impractical in two very common situations:

• The measurements are very few, so the system has many -possibly infinite- solutions.

• real measurements do not *exactly *satisfy the constraints due to measurements (and modelling) errors. Therefore, no solution will be found. (For instance, an unfeasible set results with the constraint *v*_1 _= *v*_2 _and the measurements {*v*_1 _= 0.5, *v*_2 _= 0.5001}.)

Hence, the approach needs refinements to deal with a lack of measurements and to introduce the "possibility" of sensor errors and imperfect models. As shown below, such difficulties can be overcame by the introduction of slack variables and a cost index, enabling a grading of the different candidate flux vectors as more or less "possible".

##### Possibilistic description of measurements

Each experimental measurement  can be described by a constraint as follows:

(18)

where *e*_*m *_is a decision variable that represents the intrinsic uncertainty of the experimental measurements, i.e. the discrepancy between the actual flux *v*_*m*_, and the measured value . For convenience (see remark below), *e*_*m *_is substituted by two non-negative decision variables, *ε*_1 _and *μ*_1_:

(19)

These decision variables *δ *= {*ε*_1_, *μ*_1_} relax the basic assertion  = *v*_*m*_, conforming a possibility distribution in (, *v*_*m*_) associated to some cost index *J*_*m*_. Among different possible choices, a simple -yet sensible- one is the linear cost index:

(20)

with *α *≥ 0 and *β *= 0 (usually, if sensor error is "symmetric", *α *and *β *should be defined to be equal).

Recalling the concepts introduced t *v*_*m *_he preliminaries section, the interpretation of (19) and (20) may be: "*v*_*m *_=  is fully possible; the more *v*_*m *_differs from , the less possible such situation is". Indeed, the event *A *= {*v*_*m *_= } ≡ {*ε*_1 _- *μ*_1 _= 0} will be fully possible – as , achieved at *ε*_1 _= *μ*_1 _= 0, and then *π*(*A*) = *e*^-0 ^= 1. On the other hand, the possibility of the event *A *corresponding to *v*_*m *_being different from  – say, for instance, *A *= {*v*_*m *_=  + *ρ*} ≡ {*ε*_1 _- *μ*_1 _= *ρ*} – will be given by . For instance, with a cost index *J*(*δ*) = 5*ε*_1 _+ 5*μ*_1_, and a measurement  = 0.1, the possibility of the actual flux *v*_*m *_being *v*_*m *_= 0.2 is *e*^-5*0.1 ^= 0.6065 ("quite" possible), and the possibility of *v*_*m *_= 1.1 is *e*^-5*1 ^= 0.0063 ("almost" impossible).

##### Remark

As explained in a subsequent section, the weights *α *and *β *should be defined related to each measurement's "a priori accuracy".

##### A global cost index

Consider now a set of measurements  with its associated slack variables *δ*_1 _= (*ε*_1_, *μ*_1_),... *δ*_*m *_= (*ε*_*m*_, *μ*_*m*_), and individual cost indices *J*_1_(*δ*_1_),... *J*_*m*_(*δ*_*m*_). The corresponding constraints will be called *measurement-based constraints*, :

(21)

In order to have a possibility distribution, under the non-interactivity assumption (6), the cost index is defined as:

(22)

where ***α ***and ***β ***are row vectors of sensor accuracy coefficients and ***ε***_**1**_, ·***μ***_**1 **_correspond to stacking in vectors the artificial variables from individual constraints.

##### The Possibilistic MFA problem

At this point, we can define the possibilistic MFA (PMFA) problem by means of the cost index *J *(22) and the set of constraints :

(23)

where the decision variables ***δ ***are the actual fluxes **v **= (**v**_**u**_, **v**_**m**_), and the slack variables ***ε***_**1 **_and ***μ***_**1**_.

The cost index *J *reflects the log-possibility of a particular combination of the decision variables, that is, the log-possibility of a particular flux vector **v**.

##### Remark

The PMFA will be cast as a linear programming problem; this is the reason why the non-negative decision variables *ε*_1 _and *μ*_1 _were introduced in substitution of *e*_*m*_. However, it can be formulated using any other optimization framework. For instance, PMFA can be easily cast under a quadratic programming framework. Throughout the paper linear programming will be assumed due to its great computational performance (solvable in polynomial time). This supposes a great advantage when dealing with large-scale metabolic networks. Nevertheless, an example using quadratic programming will be described in a subsequent section to point out the flexibility of the PMFA.

##### Example 1 – Problem statement

**Figure 1:**
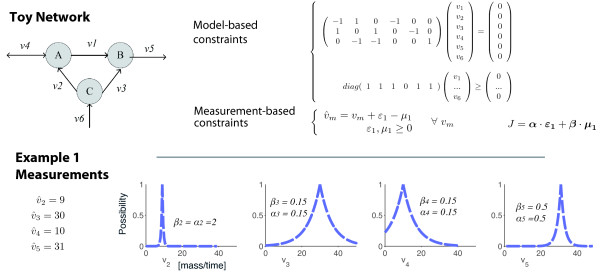
**Example 1 – Problem statement**. A toy metabolic network and the corresponding constraints are given in the top. In the bottom, a possibilistic distribution representing a set of single measurements is depicted.

Consider the toy metabolic network depicted at the top of Figure 1, and the corresponding constraints,  and . Let us consider that the measurement of *v*_2 _is "very accurate", that of *v*_5 _is moderately accurate and those of *v*_3 _and *v*_4 _are quite unreliable. The weights ***α ***and ***β ***associated to the slack variables ***ε***_**1**_, and ***μ***_**1**_, can be defined in accordance with this information: if we take *α*_2 _= *β*_2 _= 2, *α*_5 _= *β*_5 _= 0.5, and *α*_3 _= *β*_3 _= *α*_4 _= *β*_4 _= 0.15, the measurements are depicted on the bottom in Figure 1, for supposed measurements  = 9,  = 31,  = 30,  = 10.

#### Point-wise flux estimations

The simplest outcome of a PMFA problem is a point-wise flux estimation: the minimum-cost (maximum possibility) flux vector. This problem can be conveniently cast as the optimisation of a linear functional subject to linear constraints.

According to (4), the maximum possibility (minimum-cost) flux vector **v**_**mp **_corresponding to a given set of measurements is obtained as the solution to the linear programming (LP) optimization problem:

(24)

being its degree of possibility *π*(**v**_**mp**_) = *exp*(*J*_*min*_).

The obtained flux vector **v**_**mp **_contains the most possible flux values compatible (consistent) with the model and the measurements. A possibility equal to one must be interpreted as the flux vector being in complete agreement with the model and the original measurements. Lower values of possibility imply that **v**_**mp **_corresponds to fluxes **v**_**m **_deviated from the measurements .

Notice that as *π*(**v**_**mp**_) = *π*(), it can be interpreted as the "a priori" possibility of encountering the measurements ; so if it is low, this implies that either (a) there is a gross error in the measurements, (b) there is an error in the model, or both. Therefore, the maximum possibility can be used to evaluate consistency and detect errors (inconsistencies between data and models). We will come back to this point in a subsequent section.

##### Example 1, continued

Consider again the model and the measurements given in Figure 1. The maximum possibility flux vector resulting from (24) is **v**_**mp **_= (0.75, 9, 30.25, 8.25, 31, 39.3), with a possibility of *e*^-0.3 ^= 0.74. The most possible flux vector being not fully possible (peak value not equal to 1) indicates that the measurements and the model are not in complete agreement. Indeed, as a matter of fact, the model says that *v*_2 _- *v*_4 _= *v*_5 _- *v*_3_, but  = -1 and  = 1. Should the measurements had been fully compatible with the constraints imposed by the metabolic network – i.e.  = 10,  = 30,  = 30 and  = 10 – the maximum possibility flux vector would have been **v**_**mp **_= (0, 10, 30,10, 30, 40), with a possibility of *π*(**v**_**mp**_*c*) = 1.

Notice also that the possibility depends on the reliability associated to each measurement. For instance, if all the measurements were supposed to be more reliable – say *α*' = 10·*α *and *β' *= 10·*β *– the possibility distribution functions would be narrower. The interpretation of the new coefficients would, therefore, be that the same deviation from the fluxes of maximum possibility will be now be considered as a less possible fact.

### Possibility distributions as flux estimations

Clearly, a point-wise flux estimation is limited in a situation where multiple flux values might be reasonably possible. To face these situation, marginal and conditional possibility distributions (and intervals) can be obtained, again, by solving linear optimisation problems. These provide a much more informative flux estimation than a point-wise one, such as the maximum possibility flux vector, or the interval of minimum-maximum possible values in [38].

**Figure 2:**
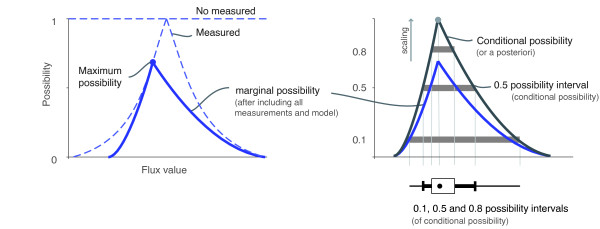
**Possibilistic flux estimations**. (Left) the figure shows possibilistic distributions representing the original single measurements, and the maximum possibility flux estimation and the distribution of marginal possibility given by PMFA. (Right) the figure shows the distributions of marginal and conditional (a posteriori) possibility. The flux intervals for conditional possibilities of 0.8, 0.5 and 0.1, and the maximum possibility estimation, are also depicted in a box-plot chart.

These flux estimations, which are illustrated in Figure 2, wil be presented along this subsection.

#### Marginal possibility distributions

Marginal possibility distributions (2) can be easily plotted and give a valuable information for the end user: they show, and rank, the possible values for each flux in the network.

The possibility of *v*_*i *_being equal to a given value *f*, *π*(*v*_*i *_= *f *∩ ), is computed by simply adding a constraint to (24):

(25)

Hence, plotting the marginal possibility for a range of fixed given values *f *(taken within a pre-specified range) will provide the marginal possibility distributions that be interpreted as the "distribution of the possible values for each flux in the network, given the measurements" (see Figure 2, left).

Notice that "cuts"  of a possibility distribution, containing those values of *v*_*i *_with a marginal possibility higher than *γ *can be obtained solving two LP problems:

(26)

This provides a highly efficient procedure to compute a possibility distribution: compute "cuts" of possibilities between 0 and 1, say, 0.1, 0.2, etc. (computing the marginal possibility of all the fluxes in the network by means of a grid of points is linear in the number of grid points and polynomial in the number of fluxes). This approach is better than defining a range of values *f *and computing its possibility with (25) because it avoids the problem of determining the most convenient step size and bounds of the flux (which, usually, are not known a priori).

#### Conditional possibility distributions

Using the definition given in the preliminaries (12), the conditional possibility distribution of a flux *v*_*i *_can be computed as follows:

(27)

Remember that conditional distribution can be obtained normalising the marginal possibility distribution to a maximum equal to one (see Figure 2).

Conditional possibility may be understood as an *a posteriori *possibility: *π*(*v*_*i *_= *f*| ) is the possibility of *v*_*i *_having the value *f*, if we assume that  is actually certain, i.e. that the model and the measurements are correct.

#### (A posteriori) Possibilistic intervals

In analogy to (26), the interval of flux values  with a degree of conditional (a posteriori) possibility higher than can be obtained solving two LP problems:

(28)

The upper bound would be obtained by replacing minimum by maximum.

These possibilistic intervals have a similar interpretation to "confidence intervals" ("credible intervals") in Bayesian statistics, providing a concise flux estimation that can be represented by means of a box-plot chart (see Figure 2, right).

##### Example 1, continued

**Figure 3:**
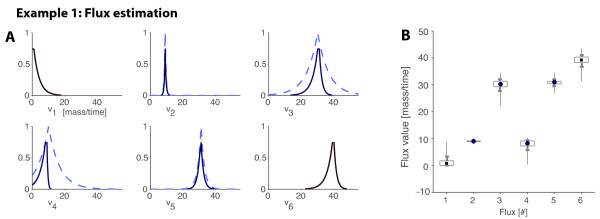
**Example 1 – Flux estimation**. PMFA estimations were obtained for the example described in Figure 1. (A) The measured values are depicted with dashed lines. The computed possibility distributions are depicted with solid lines. (B) The Figure shows the flux intervals of conditional possibility 0.8 (box), 0.5 (thick line) and 0.1 (narrow line) and the maximum possibility flux estimation (square and circle for non-measured and measured fluxes, respectively).

With the measurements in Figure 1, the resulting marginal possibility distributions for each flux are plotted in Figure 3A. They show that, for instance, the most possible value of *v*_1 _is 0.75 (possibility of 0.74), that *v*_1 _being 2.25 is quite possible, but that *v*_1 _bigger than 10 is almost impossible (possibility lower than 0.05). The possibility distributions also reflect the reliability of the estimation of each flux: the estimation of *v*_6 _is less reliable than the one of *v*_1 _or *v*_2_, since it has a wide range of highly possible values.

Notice too that the uncertainty on the measurements is often strikingly reduced through the flux estimation. For instance, the estimation of *v*_4 _– whose measurement was quite unreliable a priori – has been significantly improved, once model constraints and other measurements have been incorporated. This reflects the already noticed fact that the metabolic network structure greatly constrains the possible values of fluxes for a given, typically small, set of measured flux values. The plots of marginal possibility can also detect multiple flux vectors with maximum possibility (possibility distribution functions with flat top). Figure 3B depicts the maximum possibility flux estimation and three possibilistic intervals by means of a box-plot chart. The intervals point out that, for instance, the highly possible a posteriori values of *v*_5 _are those in [30.75, 31] (possibility greater than 0.9) and that those in [29.5, 32] are also quite possible (possibility greater than 0.5), while those outside [27, 34.5] are almost impossible as their a posteriori possibility is lower than 0.1.

### Possibilistic MFA: Refinements

Now that the basics of the PMFA framework have been introduced, some refinements will be discussed.

#### A better description of measurement's uncertainty

The formulation used above to describe the uncertainty of the experimental measurements might be considered somehow limited in some applications.

Fortunately, it is very easy to add new slack variables, and modify the  (23) and the cost index (22), allowing to work with possibility distribution functions of different characteristics.

As an example, the constraints (29) and cost (30) below describe an interval measurement plus some possibility of outlier measurements:

(29)

and

(30)

The possibility of  is one and the possibility of the actual flux being *v*_*m *_being out of the referred interval depends on the cost index weights (*α *and *β*).

For instance, a band with possibility equal to one can be used to account for *systemic *errors in measuring a particular flux, and a couple of additional slack variables may be defined to account for the decreasing possibility of *random *errors. These kind of representation of measurement uncertainty will be illustrated in subsequent examples.

##### Remark

Notice that more slack variables can be added to achieve a more complex representation of the measured flux uncertainty. In fact, any convex representation of the log-possibility uncertainty can be approximated if a sufficient number of slack variables are incorporated (*ε*_1_, *μ *_2_, *ε*_2_, *μ*_2_,...). Details are omitted for brevity.

#### Considering uncertainty in the model structure

Until now, the model-based constraints (23) – the stoichiometric relationships, reaction's irreversibility, or any other – have been considered as hard constraints; only those flux vectors **v **that exactly satisfy them could be feasible solutions. However, these constraints can be "softened" via suitable slack variables to consider uncertain knowledge. Then, these additional slack variables may be used in a cost index to generate a possibility distribution.

Consider, as an example, an equality restriction *a *= *b*. A relaxed ("softened") version of such restriction may be written as:

(31)

with ϵ and *ν *being slack variables penalised in an optimisation index *J *= *f*(ϵ, *ν*), typically with linear cost index terms, *γ*ϵ + *τυ*, in an analogous way to the discussion on uncertain measurements.

Notice also that a "softened" inequality restriction is nothing but an equality one with no penalisation on one of the slack variables above. For instance *a *≤ *b *+ *ε *can be expressed as *a *= *b *+ *ε *- *μ *with free *μ*.

Such softened model constraints may be used to roughly incorporate imprecision in the model arising, for instance, from non-compliance with the pseudo-steady-state assumption, partial unbalance of some metabolites or uncertain yields. Although these issues require further research, let us outline some preliminary ideas below.

##### Relaxing the pseudo-steady state assumption

Equation (13) derives from the dynamic mass balance around the internal metabolites **c**, where it is assumed that  ≈ 0 and that the term *μ*·**c **is negligible (*μ *denotes the growth rate). Adding slack decision variables to (13), as it has been explained, makes it possible to relax this assumption.

#### Partial unbalance of metabolites

Sometimes, a metabolite cannot be assumed to be balanced because there are reactions producing or consuming this metabolite that have not been taken into account in the network; for instance, this is often the case for the cofactors, ATP, NADP, etc. This unknown consumption/production can be represented by means of slack variables (e.g. ϵ and *υ*) if some interval limits (e.g. ϵ_*max *_and *υ*_*max*_) are provided.

##### Uncertainty in stoichiometric yields

In some cases, the value of a yield coefficient is not exactly known. This is usually the case of the yield coefficients of lump reactions used to represent biomass synthesis. Let *v*_*r *_be the flux through a reaction with an uncertain yield *Y*_*i, r *_for the metabolite *i*. The row corresponding to this metabolite in (13) can be rewritten as:

(32)

If it is known that  and *v*_*r *_is irreversible, equation (32) can be substituted by two constraints:

(33)

(34)

However, if the flux *v*_*r *_is reversible, inequalities in (33) cannot be set up, and the approach is no longer applicable. Integrating modal interval arithmetic [46] in the proposed framework might be a possible option, under research at this moment.

### Illustrative examples of other features of PMFA

Other features of Possibilistic MFA (PMFA) will be briefly illustrated using the simple metabolic network in Figure 1.

#### Example 2: Errors detection in measurements and model

As earlier mentioned, the value of the peak possibility in the resulting flux distribution provides an indication of the agreement between the model () and the measurements (). A low degree of possibility means that the model and the measurements are inconsistent. That is, that there is not any flux vector "near" the measured values satisfying the model-based constraints. Therefore, if the the maximum possibility flux vector has a low value, one must assume that either (a) there is an important error in one or more measurements, (b) there is a relevant error in the model (e.g. a mass balance is not closed, or a metabolite is not at steady state), or both.

If a high inconsistency (low possibility) is detected, it is possible to investigate what is causing it, and thus correct the measurements or improve the model. Following a straight approach, we can remove one measured flux at a time and perform the flux estimation to determine if the removed measurement was causing the low possibility. If this is the case, we may consider the following alternatives: (a) consider that  is a totally unreliable measurement and, thus, accept the flux estimation inferred from the others measurements, (b) obtaining either  again, or a different measurable flux which could provide additional information, (c) consider  a reliable piece of data and, hence, conclude that there is an error in the model or its assumptions. In case (c), a similar approach can be used to investigate which particular model-based constraint is causing the low possibility – by "softening" the suspicious constraints one at a time.

**Figure 4:**
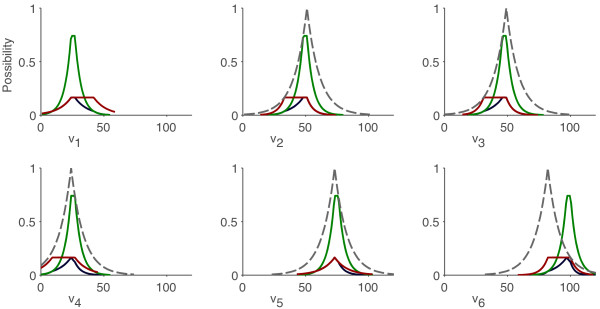
**Example 2 – PMFA to detect errors in measurements and model**. The metabolic network depicted in Figure 1 is used, assuming that five fluxes have been measured: *v*_2_, *v*_3_, *v*_4_, *v*_5 _and *v*_6 _(dotted line). The possibility distributions for each flux are depicted in three cases: using all the measurements (deep blue), removing the flux  (red) and removing the flux  (light green).

A simple example of the procedure just described is shown in Figure 4. Initially, a PMFA estimation using all the measured fluxes was performed, obtaining a maximum possibility flux vector with low possibility, *π*(**v**) = *π*() = 0.15. If the estimation is repeated removing the flux , the maximum possibility does not increase; however, when the estimation is performed removing , the maximum possibility is significantly higher (0.7). This suggests that there was a large error in the value , or an error in the model related to the balance around metabolite C which involved fluxes *v*_2_, *v*_3 _and *v*_6_.

#### Example 3: Scenario lack of data

One of the features of PMFA is that it can be used even if there is a lack of measurements; i.e. even if (14) is underdetermined or not redundant [37].

**Figure 5:**
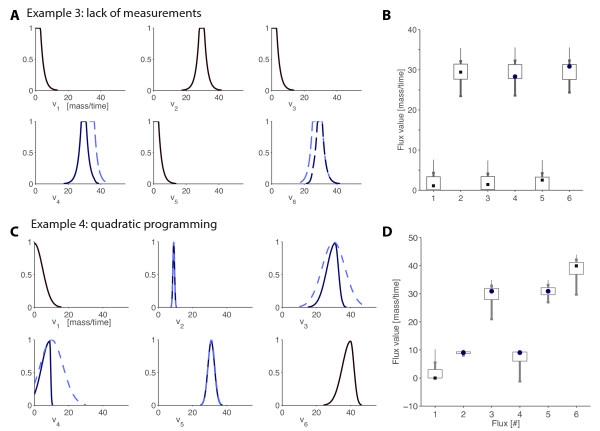
**Examples 3 and 4**. Both examples use the simple model described in Figure 1, assuming that some fluxes are measured (dashed lines). (A) (C) Possibility distributions of measured and non-measured fluxes (solid line). (B) (D) Flux intervals for conditional possibilities of 0.8 (box), 0.5 (thick line) and 0.1 (narrow line) and the maximum possibility flux estimation (squares and circles for non-measured and measured fluxes, respectively).

To point this out, let us continue with our example assuming now that only two fluxes are measured (an under-determined case). PMFA flux estimations, the marginal possibility distributions and the a posteriori intervals, are shown in Figure 5. Notice that crisp flux estimations will only be obtained if the irreversibility constraints – or other inequalities – are able to "bound" the underdeterminacy of (14). Interestingly, our experience show that this is often the case for medium size networks. Moreover, if this is not the case, the possibilistic flux estimation will be less precise – large intervals and "wide" distributions – but still reliable, i.e. the estimation will always be as precise as allowed by the available measurements and knowledge.

#### Example 4: Using quadratic programming

To show how PMFA can be cast within other optimisation frameworks, an example using quadratic programming will be discussed. We define  as  = **v**_**m **_+ **e**_**m **_and *J *= ·**W**·**e**_**m**_, where **W **is a diagonal matrix of weights. Hence, we have  for each measurement, i.e. measurements uncertainty is represented as a quadratic possibility distribution.

Let us continue with our example using the measurements of Figure 1, but representing them with the quadratic formulation just introduced. The original possibility distribution of single measurements (dashed lines) and the possibility distributions computed with PMFA (solid lines) are depicted in the Figure 5. Notice that results are similar to those obtained in the previous example (Figure 1), where the standard linear programming framework was used (even if additional auxiliar variables ϵ_2_, *μ*_2_, etc. were not used). However, the qualitative similarity between the results makes the author think that, in most cases, the linear programming setup is expressive enough and much more efficient than quadratic or other more complex optimization cases.

#### Example 5: Comparison with other methods

This example compares PMFA with traditional MFA and some of its extensions. We continue using the network depicted in Figure 1, and perform the estimations with PMFA, Traditional MFA (TMFA), MFA as a constraint, least-squares problem (LS-MFA) and the flux spectrum (FS-MFA).

**Figure 6:**
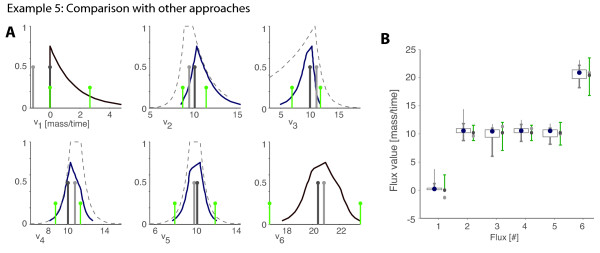
**Example 5 – Comparison of PMFA and alternative methods**. We use the model described in Figure 1 assuming that *v*_2_, *v*_3_, *v*_4 _and *v*_5 _have been measured (depicted in grey). The flux estimation was performed with four methods: PMFA, Traditional MFA (TMFA), MFA as a constraint weighted least-squares problem (LS-MFA) and the flux spectrum approach (FS-MFA). The marginal distribution computed with PMFA are depicted in blue, the point-wise estimation of TMFA and LS-MFA are depicted in light and dark grey respectively, and the interval estimation of FS-MFA in green. In (B) the maximum possibility flux estimation and the flux intervals for conditional possibilities of 0.8 (box), 0.5 (thick line) and 0.1 (narrow line) are compared with the estimations given by TMFA, LS-MFA and FS-MFA.

To show that PMFA is able to represent the measurements in a flexible way, we assume that errors in *v*_2 _and *v*_3 _are non-symmetric, and we add a band of uncertainty to account for systemic errors (Figure 6).

Inconveniently, errors have to be approximated with a normal distribution so that TMFA and LS-MFA can be used (see preliminaries). For the estimations with FS-MFA we represent the measurements with the interval of 95%, or 2*σ *(see [38]). All the results are depicted in Figure 6. Notice that TMFA assigns a negative value to an irreversible flux, *v*_1_, since it is not taking these constraints into account – this was predictable, but it must be highlighted because TMFA is still being widely used in the literature. The results also point out that the possibilistic distribution (and intervals) are much more informative than the point-wise estimations of TMFA and LS-MFA, or the intervals of FS-MFA. Basically, point-wise estimations fail when several flux values reasonably possible, whereas the flux spectrum interval tend to be conservative. Furthermore, remember that TMFA and LS-MFA cannot be used in scenarios lacking data, such as example 3, where PMFA was shown to be valuable.

To complete this perspective, the next section will discuss a comparison between PMFA and Monte Carlo approaches.

#### Example 6: Comparison with Monte Carlo

**Figure 7:**
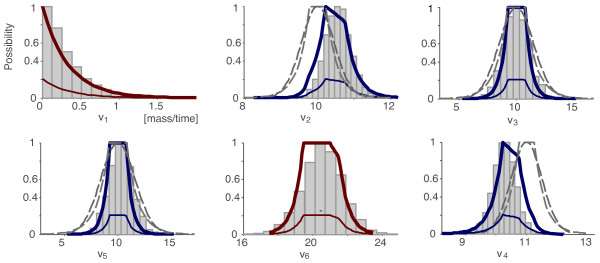
**Example 6 – Comparison of PMFA and Monte Carlo methods**. We use the simple model described in Figure (3) assuming that *v*_2_, *v*_3_, *v*_4 _and *v*_5 _have been measured. (1) PMFA: the measurements represented in possibilistic terms using linear terms are depicted in grey, and the possibility distributions calculated from them in blue (thin lines for marginal distributions and thick lines for conditional ones). (2) Monte Carlo approach: the measurements represented assuming that errors are normally distributed are depicted in grey and the histograms are those resulting from the Monte Carlo simulations.

Continuing with our example, now measurements are represented (a) in possibilistic terms (linear case) and (b) with a "similar" probabilistic formulation assuming that errors are normally distributed. Both representations are depicted in Figure 7 (dashed lines). Then, we performed two flux estimations using (a) PMFA and (b) Monte Carlo simulations (1.7 millions of combinations of values of measured fluxes were generated, taken into account their normal distribution). The conditional possibility distributions and the histograms resulting from PMFA and Monte Carlo, respectively, are depicted in Figure 7. Even if probability and possibility are not truly equivalent, a reasonable similarity between the results from both approaches exists.

Notice also that this is a simple case where Monte Carlo can be applied. Nonetheless, its worst performance is clear: the cost of computing the possibility distributions is polynomial in the number of fluxes (as shown above), whereas the cost of a Monte Carlo approach grows exponentially with the number of independent decision variables.

#### Larger-scale Example: C. glutamicum

In this section we apply Possibilistic MFA (PMFA) to a medium-size example. For illustrative purposes, we have chosen a very well-know metabolic model of *Corynebacterium glutamicum*.

##### Metabolic network model

The metabolic network of *C. glutamicum *has been taken from [47] and is a slight variation of the one originally constructed in [48,49]. The reactions considered in describing the biochemistry of the primary metabolism of *C. glutamicum *necessary to support lysine and biomass synthesis from glucose are given in the additional file 1. A reaction of ATP dissipation is included in the network, so that the ATP balance could be maintained, without actually constraining the flux space. On the contrary, the co-factors NADP, NAD and FAD are supposed to be balanced. The reaction for biomass formation is an approximation using as reactants those amino acids that explicitly appear in the network and the precursors of the other amino acids synthesized by *C. glutamicum*.

###### PMFA setting

The stoichiometric relationships, embedded in a 36 × 40 stoichiometric matrix, and the irreversibility of certain reactions, embedded in a 40 × 40 diagonal matrix, define our model-based constraints () according to (17). Both matrices are given in the additional file 1.

###### Experimental measurements

**Figure 8:**
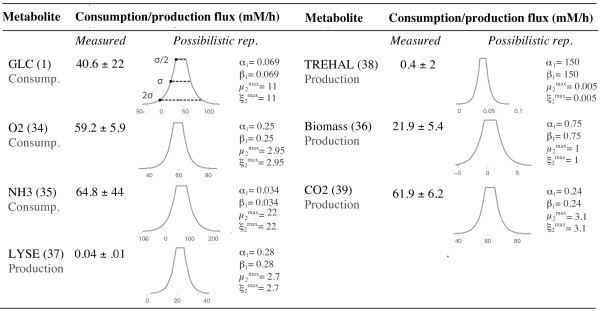
**Experimentally measured fluxes during a batch fermentation of C. glutamicum**. The second column contains the experimental measurements and their standard deviation taken from [48]. The possibility distribution representing each single measurement is depicted in the third column, and the used weights are given in the last one.

Experimental data of a batch fermentation of *C. glutamicum *cultured on minimal glucose medium has been taken from [48]. There, the growth rate and the fluxes (production/consumption rates) of the external metabolites – lactate, acetate, glucose, O2, CO2, NH3, lysine and trehalose – were experimentally measured. Since the accumulation of lactate and acetate was negligible, their flux is always zero in this case study. The measured fluxes *v*_*GLC *_(1), *v*_*O*2 _(34), *v*_*NH*3 _(35), *v*_*LY *_(37), *v*_*Thre *_(38) and *v*_*CO*2 _(39) and the growth rate *v*_*Bio *_(36), and also their standard deviations, are given in Figure 8.

###### PMFA setting

Using the data in Figure 8, we have built a possibilistic representation of single measurements defining convenient auxiliar variables and weights. The criterion to choose the weights was: full possibility for *v*_*m *_∈  ± *σ*/2 and possibility 0.5 for those in ± *σ*. The values in ± 2·*σ *have possibility 0.1 (*σ *denotes standard deviation. If errors are assumed to be normally distributed, these levels correspond to the probabilistic confidence intervals of 38%, 68% and 95%, respectively). The resultant decision variables and weights define our measurement-based constraints () according to (21). These possibilistic representations are depicted in Figure 8.

##### Possibilistic flux estimation of C. glutamicum

We used all the available measurements – *v*_*GLC *_(1), *v*_*O*2 _(34), *v*_*NH*3 _(35), *v*_*LY *_(37), *v*_*Thre *_(38), *v*_*CO*2 _(39) and *v*_*Bio *_(36) – to obtain the maximum possibility flux vector (results given in the additional file 1). The flux vector had a degree of possibility 0.38, which could be considered "low" if one considers that a significant degree of uncertainty was already being taken into account (table 1). We then obtained the marginal possibility distributions for each flux, which inspection indicated that the low possibility was almost completely caused by only one measured flux, *v*_*NH*3 _(35). This suggests that this measurement was inaccurate, or that its standard deviation was underestimated. Interestingly, this flux was indeed the most uncertain one in the original dataset (its standard deviation was a huge 44 mM/h for a nominal value of 64.8 nM/h).

**Figure 9:**
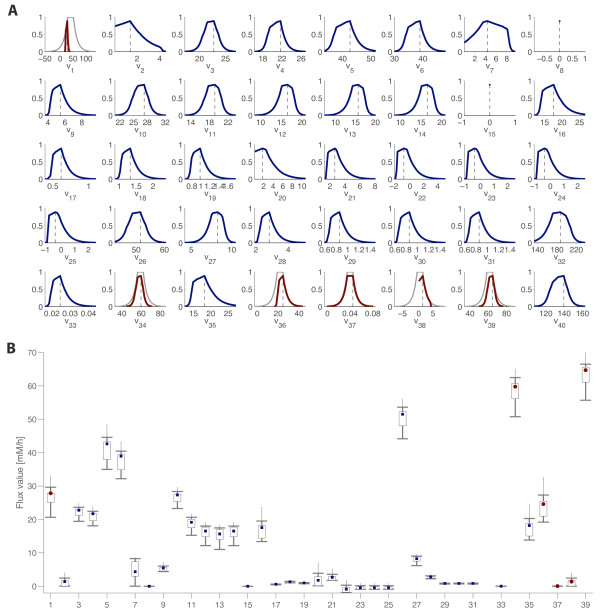
**Possibilistic flux estimation for C. glutamicum**. The measured fluxes are *v*_*GLC *_(1), *v*_*O*2 _(34), *v*_*NH*3 _(35), *v*_*LY *_(37), *v*_*Thre *_(38) and *v*_*CO*2 _(39) and *v*_*Bio *_(36). (A) Marginal possibility distributions for each flux are depicted. The original distribution of single measurements appear in grey (thick line). (B) The maximum possibility flux estimation (circles and squares for measured and non-measured fluxes, respectively) and the flux intervals for conditional possibilities of 0.8 (box), 0.5 (thick line) and 0.1 (narrow line) are depicted. All fluxes in mM/h.

As a results of this analysis – which is a rough example of the procedure mentioned in a previous section – we decided to remove the measurement and repeat our calculations. As expected, this time we obtained a maximum possibility flux vector with a similar shape, but higher possibility (0.88). The marginal possibility distributions are depicted in Figure 9A, and the maximum possibility flux estimation and the flux intervals are depicted in Figure 9B. Numerical data are given in the additional file 1, where they can be compared with those obtained if the measurement of *v*_*NH*3 _is used.

##### Possibilistic flux estimation lacking measurements

**Figure 10:**
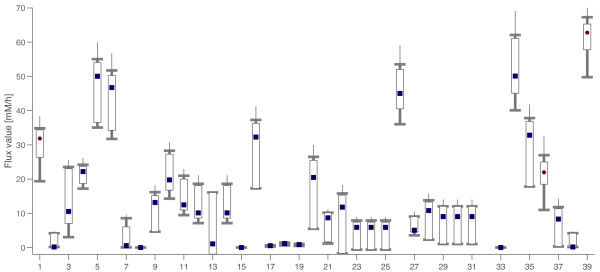
**Possibilistic flux estimation for C. glutamicum lacking measurements**. In this case the measured fluxes are only *v*_*GLC *_(1), *v*_*CO*2 _(39) and *v*_*Bio *_(36). The maximum possibility flux estimation (circles and squares for measured and non-measured fluxes, respectively) and the flux intervals for conditional possibilities of 0.8 (box), 0.5 (thick line) and 0.1 (narrow line) are depicted. All fluxes in mM/h.

We performed a flux estimation using only data of three extracellular fluxes that can be measured with standard equipment: *v*_*GLC *_(1), *v*_*CO*2 _(39) and biomass *v*_*Bio *_(36). In this case, the obtained maximum possibility flux vector is fully possible. This flux vector and the flux intervals are depicted in Figure 10. Remarkably, even if only three fluxes were measured, there was a small range of flux vectors with an a posteriori possibility higher than 0.8. Numerical results are given in the additional file 1.

##### Possibilistic flux estimation with uncertain model

As explained above, we can "soften" the model-based constraints to relax the pseudo-steady state assumption. As example, we assumed a degree of uncertainty around all the mass balances introducing decision variables **ϵ**_**1 **_and ***υ***_**1 **_and weights *γ*_1 _= *τ*_1 _= 2 (see Figure 8). Hence, flux vectors which imply small accumulations of some metabolites will be accepted, yet considered less possible.

It could be also stated that the metabolic network used herein, the one introduced by Vallino et al., relies on an unrealistic assumption: that co-factors NADP, NAD and FAD are balanced [50,51]. To avoid this, we can remove these metabolites from our stoichiometric matrix or, as an alternative, use the expressivity of the possibilistic framework to allow a certain degree of unbalance for these metabolites. Just as example, herein we assumed that cofactors may be unbalanced with some limits (say, 30 mM/h for NADP/NADPH and 15 mM/h for FAD/FADH and NAD/NADH). This "knowledge" can be easily incorporated into our model defining the convenient auxiliar variables and weights (as explained above).

**Figure 11:**
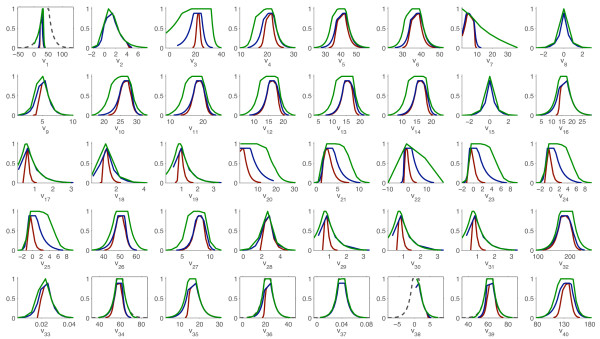
**Possibilistic flux estimation for C. glutamicum with uncertainty in the model**. The marginal possibility distributions for each flux are depicted in three cases: (a) the model-based constraints are not relaxed (red) (b) the pseudo-steady state assumption is relaxed and NADP/NADPH is allowed to be unbalanced (deep blue), and (c) the pseudo-steady state assumption is relaxed and the three cofactors – NADP/NADPH, FAD/FADH and NAD/NADH – are allowed to be unbalanced (light green). The original distribution of single measurements are depicted with dashed lines. All fluxes in mM/h.

At this point, PMFA was performed in three scenarios: (a) the model-based constraints are not relaxed (reference case) (b) the pseudo-steady state assumption is relaxed and NADP/NADPH is allowed to be unbalanced, and (c) the pseudo-steady state assumption is relaxed and the three cofactors – NADP/NADPH, FAD/FADH and NAD/NADH – are allowed to be unbalanced. The marginal possibility distributions obtained in each case are compared in Figure 11, where it can be observe how the model uncertainty is translated into the flux estimations; consider this uncertainty results in less precise flux estimations, given the less reliable model equations.

## Conclusion

In this paper we have discussed a possibilistic framework for the estimation of the metabolic fluxes shown by cells at given conditions given.

Considering ordinary constraint-satisfaction problems, metabolic fluxes fulfilling a set of model-based constraints and compatible some experimental measurements are "possible", otherwise "impossible". In this paper, this idea is refined to cope with uncertain knowledge – in the form of measurements errors or imperfect models – by introducing the notion of "degree of possibility", which enables grading the candidate flux values as more or less possible. Then, possibilistic MFA is able to query the flux vector of maximum possibility. Moreover, when multiple flux vectors might be reasonably possible, the marginal and conditional possibility distributions for each flux can be computed.

Possibilistic MFA overcomes several limitations of traditional MFA and some of its extensions. It considers measurements uncertainty in a flexible way (e.g. non-symmetric error or a band of uncertainty due to systemic error) and also model imprecision, and it is reliable even if only a few fluxes are measurable (a common scenario). Possibilistic MFA also computes possibility distributions (and intervals) which are more informative than point-wise estimations when multiple flux values might be reasonably possible. These distributions are also better than the intervals provided by the flux spectrum, or other methods giving upper and lower bounds for the fluxes. In addition, Possibilistic MFA has the ability to detect, and handle, inconsistencies between measurements and model. Finally, it must be remarked that Possibilistic MFA estimations have been cast as linear optimisation problems, for which widely-known and efficient tools exist (a MATLAB script solving example 1 is given in the additional file 2 to illustrate this point). This great computational performance makes the methodology capable of dealing with large-scale or even genome-scale metabolic networks.

It must be noticed that there is a challenge when estimating the fluxes in large-scale networks because there may be diffierent flux vectors compatible with the few available measurements [52]. Interestingly, the proposed methodology is still of use in this situation: possibilistic MFA will detect all these flux vectors that are equally possible (or even similarly possible) and depict them by means of possibilistic distributions or intervals (e.g. example 3). Unfortunately, if there is a wide range of candidates, the estimation may be sometimes little informative (but at least we can be sure that it is reliable, because all the flux vectors compatible with model and measurements are captured). One strategy to face this difficulty consists of using a rational hypothesis to promote certain flux vector among those that are equally possible. For instance, it can be assumed that cell behaviour has evolved to be optimal in some sense, so that the fluxes are optimally regulated depending on the given environmental conditions, and then invoke this principle to choose particular flux vectors [3,7,9]. There might be still alternate optima, but the approach will reduce the range of possible flux vectors. Notice that this optimality principle, or any other hypothesis, might be incorporated into the possibilistic framework as far as they are encoded in in the form of a cost index (but sometimes it will not be the case or, in other cases, its optimization will not be computationally simple). This point requires further work.

Further extension may also address the adaptation of the ideas introduced herein to metabolic flux analysis with data from labelling experiments (13C-MFA) [12-14]. Extracellular dynamics could be also taken into account incorporating measurements in different time instants [38]. Finally, we are currently developing a software that implements the Possibilistic MFA methods and its future extensions, which will be freely available for academia.

In summary, this papers introduces a unifying framework for flux estimation and (possibilistic) evaluation of consistency that is flexible, usable in scenarios lacking data, highly informative, and computationally efficient. In our opinion, the combination of computational efficiency and flexibility of the assumptions is a distinctive advantage with respect to other approaches which either may rely on stronger assumptions (chi-squared distributions, interval-only descriptions, absence of irreversibility), or be only data-based (so they do not incorporate, say, stoichiometric model balances), or provide only point-wise estimates (of flux or consistency), or be computationally intensive (multi-variate integration in a general Bayesian estimation problem).

## Authors' contributions

FLL, AS and JP designed the study, analyze the results and conceptualized the manuscript. FLL performed the estimations of the case studies. All authors read and approved the final manuscript.

## Supplementary Material

Additional file 1 **Realistic example**. Additional material related with the experimental case study with C. glutamicum. This includes a list of reactions and metabolites, the stoichiometric matrix and measurements data. Numerical results of the four examples described in the article are also included. Click here for file

Additional file 2 **Code for solving a simple example**. Simple MATLAB script solving example 1; it computes the maximum possibility flux vector and the marginal possibility distribution for *v*_6_. Format: Matlab Script (m). Further details are given in Click here for file
